# Pediatric Eye Health‐Seeking Behavior in Ghana: A Community‐Based Cross‐Sectional Study

**DOI:** 10.1002/hsr2.72179

**Published:** 2026-04-11

**Authors:** Albert Kwadjo Amoah Andoh, Nana Akwasi Owusu Mensah, Eunice Acheampong Boadu, Josephine Ampomah Boateng, Chelsea Charlyne Kosoku, Isaiah Osei Duah Junior, Werner Eisenbarth, Kwadwo Owusu Akuffo

**Affiliations:** ^1^ Department of Optometry and Visual Science, College of Science Kwame Nkrumah University of Science and Technology Kumasi Ghana; ^2^ Department of Education and Psychology, College of Educational Studies University of Cape Coast Ghana; ^3^ Department of Psychology, College of Health and Human Sciences North Carolina Agricultural and Technical State University Greensboro NC USA; ^4^ Department of Applied Science and Mechatronics HM Hochschule München University of Applied Sciences Munich Germany

**Keywords:** child, childhood visual impairment, Ghana, health care utilization, health service accessibility, health‐seeking behavior

## Abstract

**Background and Aims:**

Childhood visual impairment remains a major public health concern in low‐and‐middle income settings, including Ghana, where timely intervention can prevent avoidable vision loss. Although Ghana's National Health Insurance Scheme (NHIS) aims to reduce financial barriers, pediatric eye health‐seeking behavior among caregivers is poorly understood. This study assessed determinants of pediatric eye care–seeking behavior across multiple districts in Ghana.

**Methods:**

A community‐based cross‐sectional study was conducted among 622 children aged ≤ 20 years and their caregivers in selected Ghanaian districts. Structured questionnaires collected data on socio‐demographic characteristics, NHIS coverage, prior eye examinations, and eye care–seeking practices. Multivariable logistic regression was used to identify factors associated with seeking eye care (*p* < 0.05).

**Results:**

Among caregivers who reported that their child had experienced an eye problem (60.3%, 375/622), fewer than half (41.2%, 154/374) sought professional eye care, most commonly at general health facilities rather than specialized eye clinics. In multivariable analysis, children residing in peri‐urban (AOR = 5.50, 95% CI: 1.33–23.47; *p* = 0.02) and urban districts (AOR = 6.91, 95% CI: 2.13–22.38; *p* = 0.001) had significantly higher odds of seeking eye care compared with those in rural areas. Prior eye examination history also influenced care‐seeking: children examined within the past 2 years had lower odds of seeking additional eye care (AOR = 0.01, 95% CI: 0.00–0.04; *p* < 0.001), whereas those whose last examination occurred ≥ 2 years previously had higher odds of seeking care (AOR = 3.73, 95% CI: 1.18–11.82; *p* = 0.025), relative to children who had never undergone an eye examination. Socio‐demographic factors, including education, income, and insurance status, showed no significant associations.

**Conclusion:**

Overall, the study identified limited utilization of pediatric eye care services that is influenced by settlement type and previous engagement, rather than socioeconomic status. Strengthening community‐based outreach and embedding pediatric eye care into primary healthcare can enhance early intervention and reduce avoidable childhood visual impairment.

## 1 Introduction

Pediatric eye care‐seeking behavior represents a critical yet understudied determinant of childhood visual health outcomes in low‐and‐middle income countries (LMICs) [1, 2] The consequences of delayed or absent care extend beyond vision loss to significantly impair children's educational participation, social development, and overall quality of life [3, 4], while timely interventions such as cataract surgery demonstrably restore independence and improve family well‐being [5]. Despite effective interventions, childhood visual impairment (CVI) remains a major public health challenge, especially in LMICs [6]. In Africa, utilization is hindered by geographic inaccessibility, high costs, workforce shortages, and low parental awareness [2, 7].

Access to timely pediatric eye care in LMICs remains limited, allowing preventable conditions to progress to irreversible vision loss [8, 9]. Caregiver health‐seeking behavior strongly shapes the burden, yet many LMIC health systems prioritize infectious diseases over pediatric eye disorders due to limited resources [10]. Parents and guardians act as gatekeepers, with decisions shaped by perceived severity, financial capacity, trust in the health system, and knowledge of available services [11].

Ghana, with an estimated population exceeding 30 million [12], has a predominantly young demographic structure, yet access to specialized pediatric eye care service delivery is severely constrained by uneven healthcare infrastructure and highly skewed distribution of eye care professionals across regions [13, 14]. In the Ashanti region, for instance, pediatric eye care services are available in 90% of health facilities, but disparities exist due to limited continuing education for professionals and inadequate resources [13]. While national data on CVI are scarce, available evidence suggests that avoidable and treatable conditions, such as uncorrected refractive errors, amblyopia, and childhood cataract, contribute to over 87% of cases, with 49.2% being treatable and 38% preventable [15].

Compounded by these factors, families face long travel distances, financial constraints, and low awareness of pediatric eye conditions [16, 17]. Although the National Health Insurance Scheme (NHIS) was introduced to reduce financial barriers to health care in Ghana, its effectiveness in promoting pediatric eye care is unclear [18]. Most studies in Ghana focus on adults or facility‐based settings, leaving a gap in community‐level evidence on children [11, 19, 20, 21, 22, 23]. Moreover, little is known about how socio‐demographic factors interact with systemic issues, including insurance coverage, to shape caregiver decisions [1].

This study examines determinants of pediatric eye health‐seeking behavior in Ghana, particularly assessing socio‐demographic influences, barriers to service use, and the role of NHIS coverage. By integrating behavioral and health system perspectives across multiple districts, it offers novel insights into drivers and constraints of care utilization. Findings aim to guide strategies that strengthen health systems, reduce inequities, and advance universal eye health coverage to improve visual outcomes for children in resource‐limited settings.

## 2 Methods

### 2.1 Study Design, Population, and Setting

A cross‐sectional study was conducted in the Ashanti Region, Ghana's second most populous region [12]. Districts were stratified by urbanization, literacy, economic activity, and population density into four categories: highly urbanized, peri‐urban, mixed rural–urban, and predominantly rural. One district was purposively selected from each stratum, Oforikrom Municipal, Afigya Kwabre South, Atwima Kwanwoma, and Bosome Freho, based on child population size and gender balance. To enhance representativeness, Juaben Municipal, a peri‐urban district, was randomly added, reflecting the region's 61.6% urban/peri‐urban population.

### 2.2 Study Participants and Eligibility Criteria

Participants were children and adolescents aged 0–20 years, encompassing early childhood through late adolescence, during which eye care–seeking decisions are commonly caregiver‐mediated in the Ghanaian context. Respondents with incomplete demographic or clinical data were excluded. Eligibility required continuous residence in the study community for ≥ 6 months, parental/guardian consent, and absence of major cognitive or communication limitations. Visual impairment was defined as presenting visual acuity of 6/18 or worse, per ICD‑11 criteria [24].

### 2.3 Sampling and Sample Size

A stratified purposive sampling strategy was used to select study sites and participants. The minimum sample size of 384 was calculated using the standard formula for cross‑sectional studies (*n*=*Z* ^2^⋅*p*(1 − *p*)/*e* ^2^), assuming a 95% confidence level, a population proportion estimate of 50% expressed as a decimal [21, 25], and a defined margin of error.

### 2.4 Data Collection Procedures

Data were collected over 2 months by trained investigators holding a Doctor of Optometry (OD) degree, with formal tertiary‐level training in public health, using a structured questionnaire adapted from validated tools [26]. Items were mainly close‐ended with categorical options, supplemented by open fields. Demographic data included child age, sex, education, birth position, insurance, guardian education, occupation, and district. Visual acuity was assessed at distance and near, refractive status was measured with the Plus Optix Autorefractor, and anterior/posterior segments were examined by a licensed optometrist using an ophthalmoscope.

Information collected on eye health‐seeking behavior included reported eye complaints, service utilization, care location, barriers, prior exams, diagnoses, and family history. All investigators were recruited from the study region, and questionnaires were administered in English and Twi, with responses recorded on paper and later entered into Excel. Participation was voluntary, with the option to withdraw at any time without consequence.

### 2.5 Ethical Approval

The study adhered to the Declaration of Helsinki and received ethical clearance from the Committee on Human Research, Publication and Ethics, Kwame Nkrumah University of Science and Technology, Kumasi (Approval ID: CHRPE/AP/1245/24). Authorization was obtained from district health authorities and community leaders. Written informed consent was secured from participants and guardians, and documents were translated into the local dialect or read aloud by the investigators when necessary to ensure comprehension.

### 2.6 Statistical Analysis

Data were analyzed using IBM SPSS Statistics version 27. Frequencies and percentages summarized categorical variables, while bivariate and multivariate logistic regression identified factors associated with demographics, insurance coverage, and eye health‐seeking behavior. Variables with *p* < 0.05 in bivariate analyses were included in multivariate models. Of 622 participants, valid responses varied slightly due to item non‐response, addressed through complete‐case analysis.

## 3 Results

### 3.1 Socio‐Demographic Characteristics of Participants

Of the 622 participants who completed the study, there was a nearly equal sex distribution (53.4% female, 46.6% male). Most children were aged 8–12 years (38.6%), lived in peri‐urban areas (60.9%), and attended primary school (52.1%), while 7.9% had no formal education. Guardians were generally well educated: 49.8% had secondary, 28.8% primary, and 9.4% tertiary education. The majority were married/cohabiting (72.8%), households typically had three to four children (42.6%), and employment was high (84.5%), though 62.4% earned below GH₵1000 (≈ US$87) monthly. NHIS coverage was widespread (89%). Among examined children (*n* = 569), 6.1% had visual impairment and 93.9% normal vision (Table 1).

**Table 1:** Characteristics of the study population.

Variable	*n* (%)
Sex	
Male	290 (46.6)
Female	332 (53.4)
Type of settlement	
Rural	90 (14.5)
Peri‐urban	379 (60.9)
Urban	153 (24.6)
Participants' age group in years	
0–4	144 (23.2)
5–9	221 (35.5)
10–14	210 (33.8)
15–20	47 (7.6)
Participants' education level	
No formal education	49 (7.9)
Pre‐school	153 (24.6)
Primary	324 (52.1)
Secondary	96 (15.4)
Guardians' education level	
No formal education	74 (12.0)
Primary	178 (28.8)
Secondary	308 (49.8)
Tertiary	58 (9.4)
Guardians' marital status	
Single	94 (15.2)
Married	387 (62.7)
Co‐habiting	62 (10.0)
Separated	15 (2.4)
Divorced	32 (5.2)
Widow	27 (4.4)
Number of children in household	
< 3	131 (22.3)
3–4	250 (42.6)
5–6	146 (24.9)
> 6	60 (10.2)
Child's position among siblings	
First child	146 (24.3)
Middle child	279 (46.4)
Last child	176 (29.3)
Guardians' Income	
Below 1000	329 (62.4)
1000–2999	152 (28.8)
3000–5999	36 (6.8)
6000 and above	10 (1.9)
Guardians work status	
Employed	523 (84.5)
Unemployed	83 (13.4)
Retired	13 (2.1)
Health insurance coverage	
No	68 (11.0)
Yes	551 (89.0)
Presenting visual impairment	
No	536 (93.9)
Yes	35 (6.1)

*Note: n* = frequency; % = percent frequency.

### 3.2 Eye Health‐Seeking Behavior of the Sample

Overall, 60.3% of guardians reported a child's eye complaint, yet only 41.2% sought professional care, while 58.8% did not. Among those who sought care, 44.8% visited general health facilities, 24.7% went to pharmacies or other places, and 31.8% consulted optometrists or ophthalmologists. Of those who did not seek care, 52.5% cited personal or unspecified reasons such as being too busy, perceiving the eye problem as normal or not serious, deciding not to seek care, intermittent symptoms, and delayed recognition of the problem, 29.2% reported cost barriers, 16.3% attributed it to cultural perceptions, and 3.5% faced access challenges. Prior eye examination history was limited: 66.2% of children had never been examined, 20.7% had an exam within 2 years, and 13.1% more than 2 years ago. Previous eye disease was reported in 4.2% of children, and 17.4% had a family history of eye disease (Table 2).

**Table 2:** Eye health‐seeking behavior of the sample.

Variables	*n* (%)
Has your child ever complained about their eyes	
No	247 (39.7)
Yes	375 (60.3)
	
Did you seek eye care for your child	
No	220 (58.8)
Yes	154 (41.2)
	
Place sought for eye care	
General hospital/health center	69 (44.8)
Optometrist/Ophthalmologist	49 (31.8)
Pharmacy/other	38 (24.7)
Reason for not seeking eye care	
Personal/no reason	106 (52.5)
Cost	59 (29.2)
Cultural belief/perception	33 (16.3)
Access barriers	7 (3.5)
Child prior eye examination history	
Never	343 (66.2)
< 2years	107 (20.7)
≥ 2years	68 (13.1)
Child diagnosis of eye disease	
No	596 (95.8)
Yes	26 (4.2)
Family history of eye disease	
No	499 (80.2)
Yes	108 (17.4)
Don't know	15 (2.4)

*Note: n* = frequency; %= Percentage.

### 3.3 Socio‐Demographic and Systemic Correlates of Child Eye Care‐Seeking Behavior

In bivariate analyses, urban residence (OR = 2.42, 95% CI: 1.40–4.19, *p* = 0.002) and peri‐urban residence (OR = 2.07, 95% CI: 0.99–4.31, *p* = 0.053) were associated with higher odds of pediatric eye care–seeking compared with rural residence, while children aged 10–14 years had lower odds (OR = 0.48, 95% CI: 0.23–0.98, *p* = 0.04). Previous eye examination history showed the strongest associations, with markedly reduced odds among children examined within the past 2 years (OR = 0.03, 95% CI: 0.02–0.07, *p* < 0.001) and increased odds among those examined ≥ 2 years prior (OR = 5.41, 95% CI: 1.94–15.06, *p* = 0.001).

Multivariate regression adjusted for confounders showed settlement type and prior eye examination history as the strongest predictors of pediatric eye care‐seeking. Children in peri‐urban (AOR = 5.58, 95% CI: 1.33–23.47, *p* = 0.02) and urban districts (AOR = 6.91, 95% CI: 2.13–22.38, *p* = 0.001) were significantly more likely to seek care than rural peers. Compared with children never examined, those examined within 2 years were less likely to seek care again (AOR = 0.01, 95% CI: 0.00–0.04, *p* < 0.001), while those examined ≥ 2 years ago were more likely (AOR = 3.73, 95% CI: 1.18–11.82, *p* = 0.025). Other sociodemographic factors and insurance coverage were not independently associated (Table 3).

**Table 3:** Factors associated with pediatric eye health‐seeking behavior.

Variables	Bivariate logistic regression			Multivariate logistic regression		
	OR	95% CI	*p* value	AOR	95% CI	*p* value
Sex						
Male	ref					
Female	1.49	0.98–2.25	0.06			
Type of settlement						
Rural	ref					
Peri‐urban	2.07	0.99–4.31	0.053	5.50	1.33–23.47	0.019
Urban	2.42	1.40–4.19	0.002	6.91	2.13–22.38	0.001
Age group in years						
0–4	ref					
5–9	0.58	0.27–1.24	0.16	0.36	0.08–1.67	0.192
10–14	0.48	0.23–0.98	0.04	0.32	0.08–1.39	0.128
15–20	0.65	0.32–1.30	0.22	0.41	0.10–1.69	0.22
Participant educational level						
No formal education	ref					
Pre‐school	0.48	0.20–1.11	0.086			
Primary	0.58	0.30–1.12	0.105			
Secondary	0.62	0.37–1.06	0.079			
Guardian's educational level						
No formal education	ref					
Primary	0.47	0.17‐1.29	0.143			
Secondary	1.07	0.45 – 2.52	0.883			
Tertiary	1.16	0.51 – 2.62	0.729			
Guardians' marital status						
Single	ref					
Married	0.25	0.08–0.81	0.021	0.35	0.03–3.87	0.389
Co‐habiting	0.34	0.11–1.00	0.050	0.52	0.05–5.05	0.572
Separated	0.20	0.06–0.71	0.012	0.47	0.04–5.70	0.554
Divorced	0.20	0.04–1.08	0.062	0.06	0.00–1.65	0.095
Widow	0.37	0.09–1.47	0.159	0.43	0.03–6.36	0.539
Number of children in household						
< 3	ref					
3–4	1.74	0.74–4.06	0.203			
5–6	1.12	0.50–2.50	0.787			
> 6	1.44	0.61–3.41	0.409			
Child's position amongst siblings						
First child	ref					
Middle child	1.05	0.60–1.85	0.862			
Last child	0.73	0.43–1.23	0.234			
Guardians' Income						
Below 1000	ref					
1000–2999	0.33	0.06–1.82	0.202			
3000–5999	0.41	0.07–2.38	0.322			
6000 and above	0.58	0.08–4.39	0.601			
Guardians work status						
Employed	ref					
Unemployed	0.35	0.09–1.43	0.143			
Retired	0.27	0.06–1.22	0.09			
Health insurance coverage						
No	ref					
Yes	0.79	0.39–1.57	0.493			
Presenting Visual Impairment						
No	ref					
Yes	0.66	0.27–1.63	0.37			
How long has it been since your child's last eye examination						
Never	ref					
< 2years	0.03	0.02–0.07	< 0.001	0.01	0.00–0.042	< 0.001
≥ 2years	5.41	1.94–15.06	0.001	3.73	1.18–11.82	0.025
Has your child ever had a previous diagnosis of an eye disease						
No	ref					
Yes	0.22	0.07–0.69	0.01	0.65	0.10–4.09	0.643
Has any immediate family member suffered eye problems						
No	ref					
Yes	4.16	0.91–19.10	0.067			
Don't know	3.42	0.71–16.59	0.127			

*Note:* Bivariate logistic regression was performed at a significance of p < 0.05, with variables significant in the bivariate model included in the multiple logistic regression and tested at p < 0.05.

Abbreviations: AOR, adjusted odds ratio; CI, confidence interval; OR, odds ratio.

## 4 Discussion

This study examined determinants of pediatric eye health‐seeking behavior in Ghana, focusing on socio‐demographic and systemic influences. Despite high awareness of childhood eye problems, formal service uptake was low: nearly two‐thirds of caregivers reported a child's eye issue, yet less than half sought care. Prior eye examination history and settlement type were significant predictors of pediatric eye care utilization, with children residing in urban and peri‐urban areas and those with previous eye examinations showing a higher likelihood of seeking care, whereas caregiver education, household income, and insurance coverage were not associated with utilization.

CVI remains a significant public health challenge in LMICs [6]. In Ghana, Honduras, and India, while 95% of caregivers recognize the importance of pediatric eye examinations, only 34% have ever sought screening [1, 27], highlighting a gap between awareness and action. Barriers include perceived absence of symptoms, financial constraints, logistical challenges, and misconceptions about pediatric eye conditions [2, 28, 29]. Care is often sought only when problems are overtly visible, delaying detection of refractive error, amblyopia, and cataract [5, 29]. Despite recognition of these barriers, evidence remains limited on the socioeconomic, educational, and cultural determinants of care‐seeking in LMICs, particularly Ghana [1, 2]. This study hypothesizes that parental eye health‐seeking behavior is significantly shaped by socioeconomic status, service accessibility, and cultural beliefs, with favorable profiles predicting timely utilization.

This study reveals critical gaps in pediatric eye health‐seeking behavior in Ghana. Despite broad NHIS coverage, only 41.2% of guardians sought professional care for children's eye complaints, and 66.2% had never undergone an eye examination. The NHIS, established in 2003, covers multiple eye services, including refraction, visual field testing, and cataract surgery [30, 31], yet utilization remains low. This disconnect reflects broader health system challenges where service availability, awareness, and cultural perceptions outweigh financial coverage [32]. Reported barriers, including time‐related constraints, perceptual factors, intentional delay or indecision, cost, service availability, and cultural perceptions, mirror findings across Africa [2]. These barriers highlight a critical gap between symptom recognition and perceived need for professional care in community settings, suggesting that delayed or foregone care is often driven less by structural access barriers and more by caregivers' interpretation of symptom severity and competing daily priorities, particularly when symptoms are mild or episodic. Even with NHIS support, indirect costs and perception of eye care as non‐essential hinder uptake [13]. Evidence from Nigeria, Kenya, Malawi, Zambia, and Tanzania similarly shows factors such as cost, proximity to health facilities, parental knowledge, and attitudes strongly influence care‐seeking, underscoring the need for interventions including financial coverage, awareness and education, stigma, and accessibility [33, 34, 35].

Consistent with prior evidence, our findings highlight the strong influence of geographic context on pediatric eye care utilization. Similar studies in South Asia show that remoteness, low parental education, limited empowerment of women, and lack of information reduce service uptake [36]. In Ghana, children from urban and peri‑urban districts were significantly more likely to seek care than rural peers, reflecting structural and accessibility challenges outside urban centers. Geographic barriers, uneven provider distribution, and limited service availability are well‑documented determinants of low uptake in Africa and other resource‑constrained settings [2, 7]. National data similarly confirm that peri‑urban and rural populations face disadvantages due to uneven facilities and workforce distribution [37].

Prior eye examination history showed a strong association with current care‐seeking, suggesting that earlier contact with eye services reinforces future utilization through greater awareness and confidence. In this study, children previously examined were significantly more likely to seek care than those never examined, reflecting continuity of care or need for review. This aligns with evidence that caregiver knowledge, beliefs, and prior experiences shape decisions on child eye health [1, 38]. Early engagement with eye care may enhance awareness, highlight benefits, and build trust in the health system, promoting continued use.

### 4.1 Implications for Policy and Practice

NHIS coverage alone did not ensure pediatric eye care use. Settlement type and prior service engagement were the strongest predictors, highlighting geographic disparities and the need for continuity of care. Interventions should prioritize school‐based screening, structured follow‐up, and integration into primary healthcare, supported by community education and guardian involvement. Expanding district ophthalmic services and mobile outreach could help close urban–rural gaps.

## 5 Strengths and Limitations

This study's inclusion of urban, peri‐urban, and rural districts enhanced generalizability, while stratified sampling and multivariate regression strengthened inference. Causality was limited by the cross‐sectional design, potential bias introduced by guardian self‐reports, and representativeness affected by exclusion of incomplete data, underscoring the need for longitudinal and mixed‐methods research to clarify determinants of pediatric eye care‐seeking. Because caregiver descriptions of children's eye complaints were often broad and lacked sufficient clinical detail, it was not possible to reliably categorize complaint types within the study population. As a result, this variable was not analyzed further. Future studies would benefit from incorporating simplified symptom‐based classifications or clinical verification within community surveys to more accurately characterize the spectrum of pediatric eye conditions. Some associations observed in the multivariable analysis were characterized by extreme effect sizes and wide confidence intervals. These patterns likely reflect small sample sizes within specific categories and sparse data, which can inflate odds ratios and reduce estimate precision. Consequently, while the direction and statistical significance of these associations support the findings, the magnitude of the effects should be interpreted with caution. Future studies with larger samples or longitudinal designs are needed to generate more stable and precise estimates.

## 6 Conclusion

This study provides regionally grounded evidence that pediatric eye health‐seeking in Ghana is driven more by service delivery, continuity of care, and geographic access than by socioeconomic factors alone. Reducing preventable CVI requires interventions that integrate awareness, accessibility, and continuity of care with financial coverage.

## Author Contributions

Conceptualization: Albert Kwadjo Amoah Andoh, Nana Akwasi Owusu Mensah, Kwadwo Owusu Akuffo, Josephine Ampomah Boateng.Data curation: Albert Kwadjo Amoah Andoh, Josephine Ampomah Boateng, Nana Akwasi Owusu Mensah, Kwadwo Owusu Akuffo. Formal analysis: Albert Kwadjo Amoah Andoh, Nana Akwasi Owusu Mensah, Kwadwo Owusu Akuffo. Funding acquisition: Werner Eisenbarth, Kwadwo Owusu Akuffo. Investigation: Albert Kwadjo Amoah Andoh, Nana Akwasi Owusu Mensah, Kwadwo Owusu Akuffo. Methodology: Albert Kwadjo Amoah Andoh, Nana Akwasi Owusu Mensah, Josephine Ampomah Boateng, Chelsea Charlyne Kosoku, Eunice Acheampong Boadu, Kwadwo Owusu Akuffo. Project administration: Albert Kwadjo Amoah Andoh, Nana Akwasi Owusu Mensah, Josephine Ampomah Boateng, Kwadwo Owusu Akuffo, Werner Eisenbarth. Resources: Albert Kwadjo Amoah Andoh, Nana Akwasi Owusu Mensah, Josephine Ampomah Boateng, Chelsea Charlyne Kosoku, Eunice Acheampong Boadu, Isaiah Osei Duah Junior, Kwadwo Owusu Akuffo, Werner Eisenbarth. Validation: Albert Kwadjo Amoah Andoh, Nana Akwasi Owusu Mensah, Josephine Ampomah Boateng, Chelsea Charlyne Kosoku, Eunice Acheampong Boadu, Isaiah Osei Duah Junior, Werner Eisenbarth, Kwadwo Owusu Akuffo. Writing – original draft preparation: Albert Kwadjo Amoah Andoh, Nana Akwasi Owusu Mensah, Josephine Ampomah Boateng, Chelsea Charlyne Kosoku, Eunice Acheampong Boadu, Isaiah Osei Duah Junior, Werner Eisenbarth, Kwadwo Owusu Akuffo. Writing – review and editing: Albert Kwadjo Amoah Andoh, Nana Akwasi Owusu Mensah, Josephine Ampomah Boateng, Chelsea Charlyne Kosoku, Eunice Acheampong Boadu, Isaiah Osei Duah Junior, Werner Eisenbarth, Kwadwo Owusu Akuffo. Supervision: Kwadwo Owusu Akuffo, Werner Eisenbarth.

## Ethics Statement

Ethical approval for this study was obtained from the Committee on Human Research, Publication and Ethics, Kwame Nkrumah University of Science and Technology, Kumasi, with Approval No. CHRPE/AP/1245/24.

## Conflicts of Interest

The authors declare that they have no known competing financial interests or personal relationships that could have appeared to influence the work reported in this paper.

## Transparency Statement

The lead authors, Albert Kwadjo Amoah Andoh and Kwadwo Owusu Akuffo, affirm that this manuscript is an honest, accurate, and transparent account of the study being reported; that no important aspects of the study have been omitted; and that any discrepancies from the study as planned (and, if relevant, registered) have been explained.

## Supporting information


Supporting File

## Data Availability

The datasets generated and analyzed during the current study are not publicly available due to patient confidentiality, but are available from the corresponding author on reasonable request.

## References

[1] D. Ramai , R. Elliott , S. Goldin , et al., “A Cross‐sectional Study of Pediatric Eye Care Perceptions In Ghana, Honduras, and India,” Journal of Epidemiology and Global Health 5 (2014): 133–142. 25922322 10.1016/j.jegh.2014.06.004 PMC7320493

[2] S. H. Alrasheed , Z. D. Mohamed , and M. S. Alluwimi , “Childhood Visual Impairment Causes and Barriers to Accessing Eye Care: A Suggested Approach for Africa,” African Journal of Primary Health Care & Family Medicine 16 (2024): 1–7. 10.4102/phcfm.v16i1.4556 PMC11304197 39099279

[3] T. S. S. Magakwe , R. Hansraj , and Z. N. Xulu‐Kasaba , “Impact of Vision Problems on Children's Daily Activities: Insights From a Focus Group Discussion,” F1000Research 13 (2025): 1538. 40224135 10.12688/f1000research.159464.2 PMC11986411

[4] C. Gilbert and A. Foster , “Childhood Blindness in the Context of Vision 2020: the Right to Sight,” Bulletin of the World Health Organization 79 (2001): 227–232. 11285667 PMC2566382

[5] S. Frech , A. Hopkins , A. Moanda , J. Kilangalanga , and R. F. Guthoff , “Social, Educational and Medical Aspects After Cataract Surgery of Bilaterally Blind Children in Kinshasa—Perception of Parents and Children,” Children 9 (2022): 1683. 36360411 10.3390/children9111683 PMC9688321

[6] M. J. Burton , J. Ramke , A. P. Marques , et al., “The Lancet Global Health Commission on Global Eye Health: Vision Beyond 2020,” Lancet Global Health 9 (2021): e489–e551, 10.1016/s2214-109x(20)30488-5. 33607016 PMC7966694

[7] S. Hassan Alrasheed , “A Systemic Review of Barriers to Accessing Paediatric Eye Care Services in African Countries,” African Health Sciences 21 (2021): 1887–1897. 35283961 10.4314/ahs.v21i4.47 PMC8889803

[8] K. Naidoo , J. H. Kempen , S. Gichuhi , et al., “Prevalence and Causes of Vision Loss in Sub‐Saharan Africa in 2015: Magnitude, Temporal Trends and Projections,” British Journal of Ophthalmology 104 (2020): 1658–1668, 10.1136/bjophthalmol-2019-315217. 32229517

[9] J. Steinmetz , R. Bourne , P Briant , et al., “Causes of Blindness and Vision Impairment in 2020 and Trends over 30 Years, and Prevalence of Avoidable Blindness in Relation to Vision 2020: The Right to Sight: An Analysis for the Global Burden of Disease Study,” Lancet Global Health 9 (2021): e144–e160, 10.1016/s2214-109x(20)30489-7. 33275949 PMC7820391

[10] M. J. Burton , J. Ramke , A. P. Marques , et al., “The Lancet Global Health Commission on Global Eye Health: Vision Beyond 2020,” Maori Health Research Review 9 (2021): e489–e551. 10.1016/S2214-109X(20)30488-5 PMC7966694 33607016

[11] A. Ofosu , I. Osei , M. Hagan , L. Biekro , and A. K. Awedoba , “Eye Health Knowledge and Health‐Seeking Behaviours in Ghana,” African Vision and Eye Health 77 (2018): 1–10.

[12] Ghana Statistical Service . Ghana 2021 Population and Housing Census, accessed December 17, 2025, http://s1.statsghana.gov.gh/.

[13] E. M. Akpakli , A. J. Munsamy , and N. Rampersad , “Evaluation of Primary Eye Care Services for Children in the Ashanti Region of Ghana,” African Vision and Eye Health 84 (2025): 1053. 10.4102/phcfm.v17i1.4972 PMC12506816 41055184

[14] E. K. A. Morny , S. B. Boadi‐Kusi , S. Ocansey , S. Kyei , K. Yeboah , and M. A. Mmaduagwu , “Assessing the Progress Towards Achieving ‘VISION 2020: The Right to Sight’ Initiative in Ghana,” Journal of Environmental and Public Health 2019 (2019): 3813298, 10.1155/2019/3813298. 31428165 PMC6679876

[15] A. Ilechie , S. Wanye , C. H. Abraham , et al., “Inter‐Regional Trends in Causes of Childhood Blindness and Low Vision in Ghana,” Clinical and Experimental Optometry 103 (2020): 684–692, 10.1111/cxo.13041. 31916287

[16] E. A. Frempong and D. W. van Staden, , “Accessibility of and Barriers to the Use of Eye Health Services in Kumasi Metropolis, Ghana,” African Journal of Primary Health Care & Family Medicine 16 (2024): 4270, 10.4102/phcfm.v16i1.4270. 38949439 PMC11219546

[17] A. A. Ilechie , H. Otchere , C. Darko‐Takyi , and A. C. Halladay , “Access to and Utilization of Eye Care Services in Ghana,” International Journal of Health Research 6 (2013): 7–15.

[18] N. J. Blanchet , G. Fink , and I. Osei‐Akoto , “The Effect of Ghana's National Health Insurance Scheme on Health Care Utilisation,” Ghana Medical Journal 46 (2012): 76–84. 22942455 PMC3426378

[19] S. T. Adedokun and S. Yaya , “Factors Influencing Mothers' Health Care Seeking Behaviour for Their Children: Evidence From 31 Countries in Sub‐Saharan Africa,” BMC Health Services Research 20 (2020): 842. 32894107 10.1186/s12913-020-05683-8 PMC7487813

[20] S. Merepa , P. Akowuah , A. Abazele , et al., “Barriers to Utilization of Eye Care Services in the Upper East Region, Ghana,” Advances in Ophthalmology & Visual System 7 (2017): 00240.

[21] S. Ocansey , A. Kumi‐Kyereme , K. Awusabo‐Asare , et al., “Utilization of Eye Care Services Among Ghanaian Elderly Population: Evidence From a Peri‐Urban Community,” Ophthalmology Research an International Journal 1 (2013): 89–101.

[22] A.‐K. Mohammed and A. J. Munsamy , “Ophthalmic Services Utilisation and Associated Factors in the Ashanti Region, Ghana,” Ghana Medical Journal 57 (2023): 58–66. 37576369 10.4314/gmj.v57i1.9 PMC10416273

[23] S. Ocansey , S. Kyei , B. N. Gyedu , et al., “Eye Care Seeking Behaviour: a Study of the People of Cape Coast Metropolis of Ghana,” Journal of Behavioral Health 3 (2014): 101–106.

[24] World Health Organization . ICD‐11 for Mortality and Morbidity Statistics, accessed December 17, 2025, https://icd.who.int/browse11.

[25] E. M. Akpakli , A. J. Munsamy , and N. Rampersad , “Parental Knowledge and Practices Towards Primary Eye Care Services Among Children in the Ashanti Region of Ghana,” Journal of Community Medicine and Primary Health Care 37 (2025): 54–68. 10.4102/phcfm.v17i1.4972 PMC12506816 41055184

[26] J. E. Moyegbone , “Eye Health Seeking Behavior and Its Associated Factors Among Adult Population in Mangu LGA, Plateau State, Nigeria,” Journal of Ophthalmology and Advance Research 5 (2024): 1–9.

[27] D. Ramai and T. Pulisetty , “Maternal and Caregiver Perceptions to Childhood Eye Care in Ghana,” Internet Journal of Epidemiology 11 (2013): 2–8.

[28] S. H. Alrasheed , K. S. Naidoo , and P. C. Clarke‐Farr , “Childhood Eye Care Services in South Darfur State of Sudan: Learner and Parent Perspectives,” African Vision and Eye Health 75 (2016): 1–13. 10.4102/phcfm.v10i1.1767 PMC6244194 30456975

[29] J. A. Ebeigbe , “Factors Influencing Eye‐Care Seeking Behaviour of Parents for Their Children in Nigeria,” Clinical and Experimental Optometry 101 (2018): 560–564. 27990681 10.1111/cxo.12506

[30] A. Kusi , U. Enemark , K. S. Hansen , and F. A. Asante , “Refusal to Enrol in Ghana's National Health Insurance Scheme: Is Affordability the Problem?,” International Journal for Equity in Health 14 (2015): 2. 25595036 10.1186/s12939-014-0130-2 PMC4300159

[31] National Health Insurance Authority . National Health Insurance Scheme Benefits Package, accessed December 17, 2025, https://www.nhis.gov.gh/benefits.

[32] D. Dovlo and C. Atim , 2021. The National Health Insurance Scheme in Ghana.

[33] S. T. Sherief , S. Tesfaye , Z. Eshetu , A. Ali , and H. Dimaras , “Exploring the Knowledge, Attitudes, and Practice Towards Child Eye Health: A Qualitative Analysis of Parent Experience Focus Groups,” PLoS One 18 (2023): e0293595, 10.1371/journal.pone.0293595. 37922264 PMC10624311

[34] M. Mrisho , A. Mkopi , M. Mafwiri , and F. Bakar , *Evaluation of the Integration of Eye Care for Children Into Primary Health Care System in Tanzania* (Ifakara Health Institute & Muhimbili University of Health and Allied Sciences, 2014)

[35] C. T. Evans , P. D. Lenhart , D. Lin , et al., “A Cost Analysis of Pediatric Cataract Surgery at Two Child Eye Health Tertiary Facilities in Africa,” Journal of American Association for Pediatric Ophthalmology and Strabismus 18 (2014): 559–562. 25454021 10.1016/j.jaapos.2014.08.005 PMC4268264

[36] N. Ferdausi , J. Tasnim Khan , and T. Akib Khan , “Factors Influencing Utilization of Pediatric Eye Care Services in Bangladesh: A Qualitative Study,” Acta Scientific Ophthalmology 4 (2021): 116–122.

[37] D. A. Tuoyire , L. Baatiema , D. Dwomoh , and S. Bosomprah , “Healthcare Utilization in Ghana: Insights From the 2017 Ghana Living Standard Survey,” PLoS One 19 (2024): e0306032, 10.1371/journal.pone.0306032. 38917162 PMC11198759

[38] D. Masarwa , Y. Niazov , M. Ben Natan , and D. Mostovoy , “The Role of Parental Health Beliefs in Seeking an Eye Examination for Their Child,” BMC Ophthalmology 23 (2023): 269, 10.1186/s12886-023-02994-2. 37312052 PMC10262523

