# Supplementary material for: Pediatric Eye Health‐Seeking Behavior in Ghana: A Community‐Based Cross‐Sectional Study
Source: Health Sci Rep. 2026 Apr 11;9(4):e72179. doi: 10.1002/hsr2.72179 (PMC13069588; doi:10.1002/hsr2.72179)
Supplement: Supplementary file 1 — Supporting File [file HSR2-9-e72179-s001.docx]

**Appendix**

**STUDY QUESTIONNAIRE**

**PEDIATRIC EYE HEALTH-SEEKING BEHAVIOR IN GHANA: A COMMUNITY-BASED CROSS-SECTIONAL STUDY**

**SECTION A: SOCIO-DEMOGRAPHIC CHARACTERISTICS OF PARTICIPANT**

Telephone No: Participant Code:

1) What is your age in years? ……… years

2) What is your sex?

*Tick only one box*

[ ] Male

[ ] Female

3) What is the name of your District? …………

**SECTION B: SOCIO-DEMOGRAPHIC CHARACTERISTICS OF PARTICIPANT’S GUARDIAN**

1) What is your relationship with the participant? ……………………

2) What is your sex?

*Tick only one box*

[ ] Male

[ ] Female

3) What is your age in years? ------------- years

4) What is your level of education?

*Tick only one box*

[ ] No formal education

[ ] Primary

[ ] Secondary school

[ ] University/ Tertiary

[ ] Graduate school

5**)** What is your marital status?

*Tick only one box*

[ ] Single

[ ] Married

[ ] Divorced

[ ] Widow/ Widower

[ ] Separated

[ ] Co-habiting

6) Number of children …………

7) What is your ward’s(participant) position among your children?

[ ] First Child

[ ] Second Child

[ ] Third Child

[ ] Fourth Child

Other ………..

8) Was your child born full-term or pre-term? ………..

9) What is your work status?

*Tick only one box*

[ ] Employed

[ ] Unemployed

[ ] Retired

10) What is the type of your employment?

*Tick only one box*

[ ] Self-employed

[ ] Employee

11**)** What is your occupation?

12) What is your monthly income level?

*Tick only one box*

[ ] Below GHȻ200

[ ] GHȻ 200 – GHȻ 499

[ ] GHȻ 500 – GHȻ 999

[ ] GHȻ 1000- GHȻ2,999

[ ] GHȻ3,000 – GHȻ5,999

[ ] GHȻ6,000 – GHȻ 10,000

[ ] Above GHȻ 10,000

13) Are you on health insurance?

*Tick only one box*

[ ] Card bearing (Card available for inspection)

[ ] Card bearing (Card not available for inspection)

[ ] Not registered with NHIS

14) Is your ward on health insurance?

*Tick only one box*

[ ] Card bearing (Card available for inspection)

[ ] Card bearing (Card not available for inspection)

[ ] Not registered with NHIS

15) What is your ethnicity?

*Tick only one box*

[ ] Akan

[ ] Gonja

[ ] Ga/dangbe

[ ] Ewe

State if other………………….

16) Do you smoke?

[ ] Yes

[ ] No If Yes how often (daily/weekly/occasionally) and how many sticks( )?

17) Do you drink?

[ ] Yes

[ ] No If Yes how often (daily/weekly/occasionally) and how many shot glasses ( )?

**SECTION C: EYE HEALTH-SEEKING BEHAVIOUR**

**C1. KNOWLEDGE OF VISUAL STATUS**

1. Has your ward ever complained/had a problem with their eyes or vision? [ ]Yes [ ] No

***If Yes,*** what was the specific complaint/problem?...............................................

***If No continue from question 7, if Yes continue through question 2***

**C2. SELF-EYE CARE PRACTICES AND BARRIERS**

2. Did you ever seek eye care for the vision problem in **B1**? [ ]Yes [ ] No ***If Yes continue through 3, if No continue through 5***

3. Where did you go for treatment? (Please tick all boxes that apply)

[ ] General Hospital [ ] Community Health Centre [ ] Pharmacy [ ] General practitioner

[ ] Optometrist [ ] Ophthalmologist [ ] Optician [ ] Others_____________

4. Why did you choose the place you went? …………………………………

5. Is the problem OK now? [ ] Yes [ ] No

6. Why didn’t you go somewhere (else) for treatment? (**Please tick all boxes that apply**)

[ ] Cost [ ] Discrimination [ ] Language problems [ ] Transport/Distance [ ] Service not culturally appropriate [ ] Decided not to seek care [ ] Waiting time too long or not available at the time required [ ] It is normal for eyesight to get worse [ ] Too busy [ ] Not available in Area [ ] Others________________________ (specify)

7. How long ago did you last see someone about your ward’s eyes or vision?

[ ] Less than or equal to 2 years ago [ ] Greater than 2 years ago [ ] Never

**C3. PREVIOUS DIAGNOSIS OF EYE DISEASES**

8. Have you been told by an Optometrist or an Ophthalmologist that **your ward** had any of the following eye problems? (Please answer all questions)

Yes No don’t know

a. Cataract? [ ] [ ] [ ]

b. Glaucoma or high pressure in the eye? [ ] [ ] [ ]

c. Diabetic eye disease or diabetic Retinopathy [ ] [ ] [ ]

d. Age – related macular degeneration/AMD? [ ] [ ] [ ]

e. Other __________________________ [ ]

**C4. FAMILY HISTORY OF MAJOR EYE DISEASES**

9. Have any of **your immediate family** (parents, brothers or sisters) ever suffered from any of the following eye problems? (Please answer all questions)

Yes No don’t know

a. Cataract? [ ] [ ] [ ]

b. Glaucoma or high pressure in the eye? [ ] [ ] [ ]

c. Diabetic eye disease or diabetic Retinopathy [ ] [ ] [ ]

d. Age-related macular degeneration/AMD? [ ] [ ] [ ]

e. Other __________________________ [ ]

If Yes, specify who ……………………

**C5. HISTORY OF SYSTEMIC DISEASES RELATED TO EYE DEFECT**

10. Have you ever been told by a Doctor or Nurse that **your ward** has a chronic systemic condition? [ ]Yes [ ] No If so what condition is it? ……………………………
